# SPDB: a specialized database and web-based analysis platform for swine pathogens

**DOI:** 10.1093/database/baaa063

**Published:** 2020-08-06

**Authors:** Xiaoru Wang, Zongbao Liu, Xiaoying Li, Danwei Li, Jiayu Cai, He Yan

**Affiliations:** 1 School of Food Science and Engineering, South China University of Technology, 381 Wushan Road, Tianhe District, Guangzhou, Guangdong Province 510641, China; 2 Institute for Advanced Study, Shenzhen University, No. 3688, Nanhai Boulevard, Nanshan District, Shenzhen, Guangdong Province 518061, China

## Abstract

The rapid and accurate diagnosis of swine diseases is indispensable for reducing their negative impacts on the pork industry. Next-generation sequencing (NGS) is a promising diagnostic tool for swine diseases. To support the application of NGS in the diagnosis of swine disease, we established the Swine Pathogen Database (SPDB). The SPDB represents the first comprehensive and highly specialized database and analysis platform for swine pathogens. The current version features an online genome search tool, which now contains 26 148 genomes of swine, swine pathogens and phylogenetically related species. This database offers a comprehensive bioinformatics analysis pipeline for the identification of 4403 swine pathogens and their related species in clinical samples, based on targeted 16S rRNA gene sequencing and metagenomic NGS data. The SPDB provides a powerful and user-friendly service for veterinarians and researchers to support the applications of NGS in swine disease research.

**Database URL**: http://spdatabase.com:2080/

## Introduction

The swine industry is extremely important worldwide. China is the world’s largest pork producer and raises approximately half of the global pig population (1). Swine diseases are considered a serious threat to the swine industry and have had severe socio-economic impacts. For example, up to October 2019, approximately 1.2 million pigs were culled because of African swine fever (ASF) in China, causing severe economic losses (1). Pig-raising patterns in China have been altered since the ASF outbreak, which led to the closure of nearly 60% of the country’s swine farms (1–3). Furthermore, pathogens such as the *Porcine epidemic diarrhea virus* (PEDV), *Porcine reproductive and respiratory syndrome virus* (PRRSV), *Streptococcus suis*, *Mycoplasma hyopneumoniae*, *Toxoplasma gondii* and *Taenia solium* also caused considerable economic losses in the swine industry (4–9). Rapid and accurate diagnosis is an indispensable step for the successful containment of swine disease outbreaks (10). Improvements in next-generation sequencing (NGS) technology have revolutionized pathogen detection methods for disease diagnostics. The most common applications of NGS include whole-genome sequencing (WGS), targeted NGS and metagenomic NGS (mNGS) (11). WGS is useful for characterizing isolates, predicting phenotypes, tracking outbreaks and identifying infection sources (12). Targeted NGS, which focuses on specific genes, facilitates the profiling of individual microbial communities including bacteria (13), fungi (14) and parasites (15). It is a powerful tool that can aid in the understanding of disease pathogenesis as well as in the development of novel diagnostics, therapeutics and preventative measures (13). Metagenomic NGS can detect universal pathogens regardless of the type of microbe; therefore, it can be useful in the identification of novel organisms (11). Thus, NGS has gained attention for its potential utility for the investigation of the causative agents of swine diseases, as made evident by several recent studies (16–19).

However, the application of NGS in swine disease research has several limitations in the context of data, including data storage, management and maintenance. Currently, the most common data management systems used for swine pathogen sequences are the National Center for Biotechnology Information (NCBI) (20), DNA Database of Japan (DDBJ) (21) and the European Nucleotide Archive (EMBL-ENA) (22). However, information retrieval is relatively complex and inconsistent among these resources. To support the diagnosis and epidemiological investigations of highly contagious pathogens, several pathogen-specific sequence databases have been established, such as the European Classical Swine Fever Virus Database (10), Influence Research Database (unpublished), Bovine Viral Diarrhea Virus Database (23), Foot and Mouth Disease Database (24) and PRRSV-webtool (unpublished). However, there is no integrated and specific database system containing all swine pathogen sequences. The second limitation is data analysis, particularly for researchers who lack expertise in bioinformatics. Several user-friendly tools have been designed to solve this problem, such as MG-RAST (25), Metavir 2 (26), MicrobiomeAnalyst (27), SILVAngs (28) and MPD (29). However, there is no ‘swine pathogen-specific’ NGS data analysis tool available to support NGS as a diagnostic tool in swine disease containment. The third limitation is the lack of standard operating procedures (SOPs) and standards in every step of swine disease studies that use NGS, including sampling, sequencing, data submission, data analysis and data publication. Technical variations in NGS analysis must be minimized to enable efficient data sharing, improve the comparability of studies and facilitate meta-analyses (30–32).

**Figure 1:**
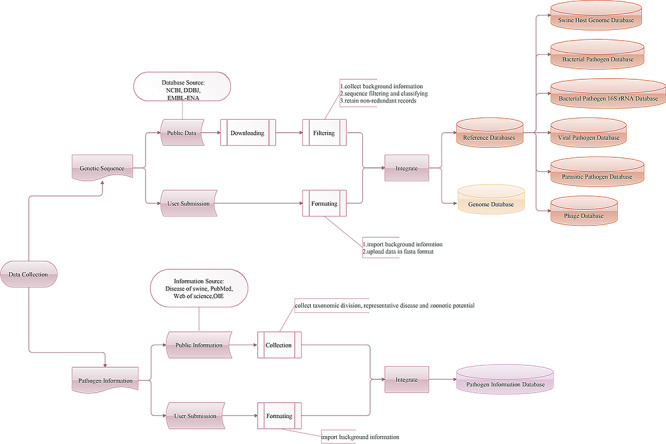
Workflow of the data integration processes. Genetic sequence and pathogen information are imported from two main sources: public resources and users.

In this study, we have developed and established the Swine Pathogen Database (SPDB) to (i) store, manage and maintain swine pathogen sequences; (ii) provide a user-friendly analysis platform for swine pathogens; and (iii) develop SOPs and standards for NGS analysis in swine disease diagnosis.

## Materials and Methods

### Database construction and implementation

The database and web interface were developed based on the LAMP technology stack. Linux (CentOS 6.10) was used as the operating system to provide maximum stability and a multithread computation environment. Apache 2.2.15 provided the most important and fundamental function as a web service. MySQL 5.5.4 was used as the relational database, and PHP 5.6.4 was adopted for dynamic web page rendering. As a web-based database, jQuery and Bootstrap were also used. Integrated analysis workflows were developed using Perl 5.10.1. The data update workflow was written in Perl 5.10.1. In this version of the SPDB, SOAP 2.21, BWA 0.7.17, Bowtie 2–2.3.5.1, Blast+ 2.6.0 and R 3.5.1 can be used for NGS analysis in web-based interactive mode. The database is scheduled to be updated on a quarterly basis.

### Database source

The SPDB contains three types of databases: pathogen information, genome and reference databases (Figure 1). Information on infectious agents affecting pigs was collected from the *Diseases of Swine* (33), a major internationally recognized reference book on swine veterinary medicine, as well as from the websites PubMed, Web of Science and the World Organization for Animal Health (OIE). Data on pathogens listed as a notifiable disease by the OIE, pathogens with a negative impact on production, pathogens with zoonotic potential and pathogens sequenced from swine-origin samples without clarified impact on the host or associated with human health were collected. Regarding parasitic pathogens, only data on endoparasites were collected. Users are also encouraged to report new pathogens. With these input data, a pathogen information database of 333 swine infectious agents, with their respective taxonomic division, representative disease and zoonotic potential, has been constructed.

The genetic sequences of swine, swine pathogens and phages associated with bacterial pathogens were imported from two main sources: public resources (NCBI, DDBJ and EMBL-ENA) and users. The SPDB supports the storage and management of data submitted directly by users. As of March 2020, the genome database of SPDB contained 18 swine host genomes, 6223 bacterial pathogen genomes, 9577 viral pathogen genomes, 5743 parasitic pathogen genomes and 4587 phage genomes. The background information of each genome is checked and rearranged. When integrating data from NCBI, DDBJ and EMBL-ENA databases, if identical projects and samples are found, the program is designed to give preference and retain the data from NCBI.

For integrated analysis workflows, in total, six reference databases have been constructed, including the swine host genome database, bacterial pathogen database (132 species), bacterial pathogen 16S rRNA database (132 species), viral pathogen database (114 species), parasitic pathogen database (87 species) and phage database (4070 species). Depending on the research strategy, the SPDB gives users the option to choose these online reference databases. The swine host genome database contains 18 swine whole genomes. The bacterial pathogen, viral pathogen and parasitic pathogen databases are non-redundant sources of bacterial, viral and parasitic pathogens, respectively. The bacterial pathogen 16S rRNA database contains only bacterial pathogen 16S rRNA sequences. The phage database is also non-redundant and collects phage sequences of bacterial pathogens. These reference databases are formatted for use with different tools.

### Validation of swine pathogen screening tool

For validation, 21 different mock communities were built and submitted to the pathogen screening tool. Each mock community contained 10 species, which were randomly selected by the shuf command from the pathogen information list. The reads datasets were built, and their abundance was calculated using wgsim software. The contig, gene, protein and 16S rRNA sequences were downloaded from GenBank. All tests were performed using the recommended parameters. We evaluated the performance of each test by examining the assignments at the species level. For each test, the predicted relative abundance or sequence number of each species was compared with the known abundance or sequence number, respectively. The Pearson correlation coefficients were calculated between predicted and known proportions for each tool and were used to evaluate the result of each test.

### Case study: accurate detection of swine pathogens from clinical samples using the SPDB

Two different datasets were chosen to illustrate the results that can be obtained with the SPDB. The first dataset contained viral metagenomic sequencing data from different piglets that died from congenital tremors (CT) in Guangdong, China. The second dataset included metagenomic data of two stillborn fetuses from a commercial swine farm in Jiangxi province. Notably, in these two fetuses, the common pathogens such as PRRSV, *Porcine circovirus 2*, *T. gondii*, *P. parvovirus*, *Classical swine fever virus*, *Japanese encephalitis virus* and *Suid alphaherpesvirus 1* had not been detected through polymerase chain reaction (PCR) tests. The nucleic acid of the collected samples was extracted using the QIAamp cador Pathogen Mini Kit (Qiagen, Hilden, Germany) according to the manufacturer’s instructions. Paired-end sequencing was performed using the Illumina HiSeq PE150 platform (Illumina, San Diego, CA), and the raw metagenomic reads were trimmed using Sickle (https://github.com/najoshi/sickle) with default parameters to remove low-quality reads. Whole-genome *de novo* assemblies were performed using SPAdes v3.13.1 (34) with the following parameters: -k 21,33,55,77 --meta --only-assembler. The metagenomic raw reads have been deposited to the NCBI Sequence Read Archive, with accession number SRP254232.

**Figure 2:**
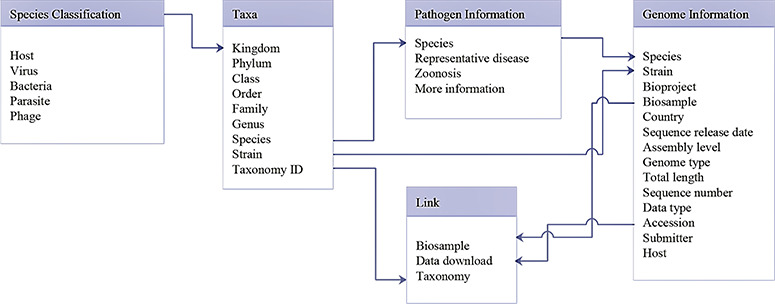
Database schema of data search and download system. Main data structure and relationships between different tables are illustrated.

## Results

### Data search and download

**Figure 3:**
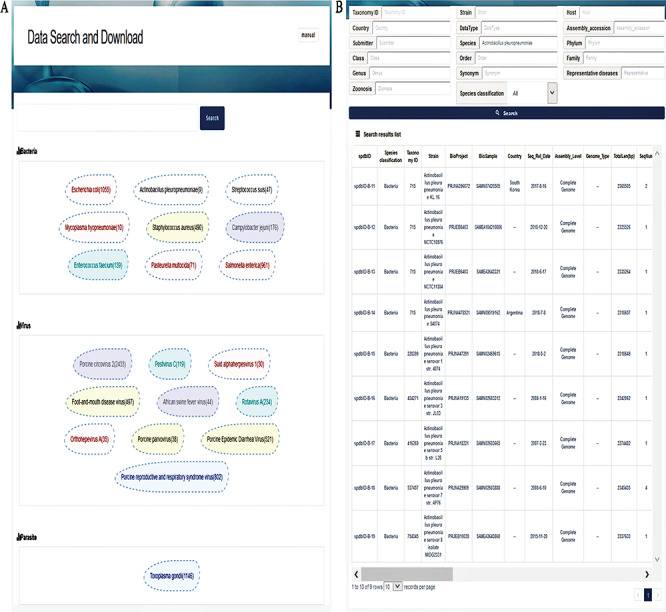
Interfaces of data search and download system. (**A**) Search page. A search box on the top is for a keyword-based search for quick and focused searches; clicking on the search without character input in the search box will allow the user to enter accurate and customized searches; the next section displays the top 20 hot words for each category, which can be directly linked to the corresponding data. (**B**) Search result page. The top of this page shows an option for advanced search, allowing the user to search the target using 17 fields to find the appropriate information more accurately. The bottom section represents the search result list, with 30 fields listed in total.

The data search and download system contains a genome database and a pathogen information database. A relational database is used to host all relevant data. A schema of the database is shown in Figure 2. The major data record types are ‘Species classification’, ‘Taxa’, ‘Genome information’, ‘Pathogen information’ and ‘Link’. ‘Species classification’ includes host, virus, bacteria, parasites and phage and is related to ‘Taxa’ by the ‘Kingdom’. Then, ‘Taxa’ could be referenced to ‘Genome information’, ‘Pathogen information’ and ‘Link’. The data search and download system is implemented by an open-source database system, MySQL. The SPDB provides three options for users to search for targets: (i) keyword-based search, (ii) ‘hot words’-based search and (iii) advanced search (Figure 3). For quick and focused searches, users can input one or more words in the keyword-based search field to obtain the desired datasets. The SPDB displays the 20 hot words in the middle of the page, and users can click these words to view the corresponding data. Based on the search records of users, hot words are recognized and added. For accurate and customized searches, an advanced search option is available, which allows the user to search the target using 17 different fields.

### Data analysis platform for swine pathogen

We designed a user-friendly data analysis platform for swine pathogens. For data protection and to explore the full functions, we recommend that users register for an account. After registering for an account, users can log into the platform, which contains five sections: personal data management, analysis process management, tasks and results management, submit data management and audit data management. In the personal data management section, users can upload their data for analysis as well as create, rename and delete files. Data stored in this part are always private. In the process management section, users can choose tools to analyze their data, manage and view job progress and download the results, which go into the tasks and results management section. The submit data management section has been designed for data updating. We encourage users to upload their data to help update the database. Users can upload different types of sequences (gene, contig, scaffold and genome) of swine pathogen in FASTA format. In the current version of the SPDB, only genome sequences will be added to both the genome database and reference database, whereas other sequences will be added only to the reference database. After uploading data, users can view the progress of data submission in the audit data management section. The system supports and accepts data with varying degrees of detail. Administrators can check and process data in the audit data management section.

**Figure 4:**
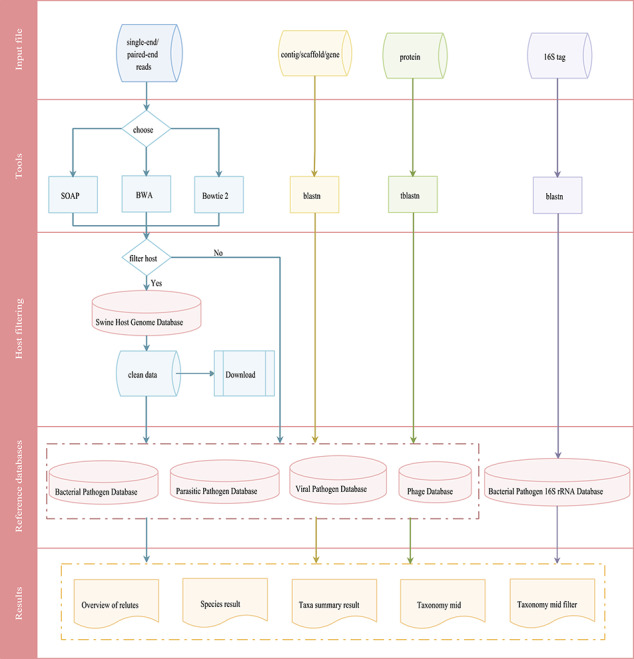
Integrated workflows for a pathogen screening tool. The workflows are shown in different colors based on input data (reads in blue, contig/scaffold/gene in yellow, protein in green and 16S tag in purple).

**Figure 5:**
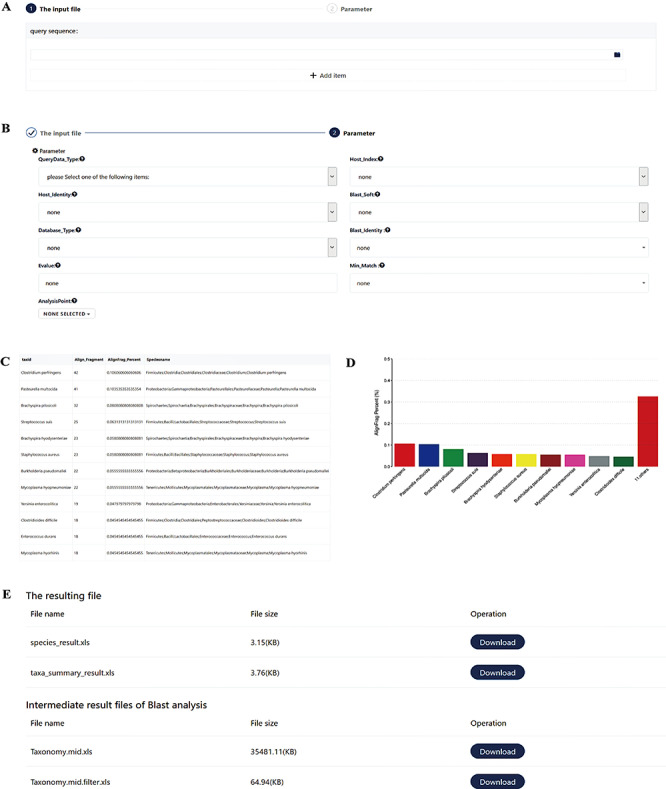
Screenshots of the pathogen screening tool. (**A**) File upload module. (**B**) Necessary arguments setting. (**C**–**D**) Overview of results. The top 10 species are shown in both a table and a histogram format. (**E**) The resulting files. The generated files can be downloaded. Species_result.xls is the result of species level. Taxa_summary_result.xls shows the result at the genus to phylum levels. Taxonomy.mid.xls contains all analysis records. Taxonomy.mid.filter.xls is the filtered result based on the set parameters and is the source file of Species_result.xls and Taxa_summary_result.xls.

### Swine pathogen screening tool

The swine pathogen screening tool is a comprehensive bioinformatics analysis pipeline for identifying 4403 swine pathogens related species in clinical samples, based on targeted 16S rRNA gene sequencing and mNGS results. Depending on the data type, the general workflow for data analysis includes two different categories that are merged in the workflow overview shown in Figure 4. The first is the metagenomic 16S rRNA sequencing amplicon taxonomic assignment, which accepts 16S rRNA data in FASTA format. Blastn is then used to align the submitted sequence against the 16S pathogen reference database. The second category is the mNGS taxonomic assignment. Depending on the research strategy, the SPDB gives users the option to choose up to four reference databases, including the bacterial pathogen database, viral pathogen database, parasitic pathogen database and phage database. For these categories, three data types are accepted: (i) reads, input files should be single-end or paired-end reads in FASTQ format retrieved from any NGS sequencer platform. An optional filtering step for host reads has been introduced to reduce the dimensionality of the input dataset and to reduce the computational analysis time. After the host contamination removal process, users can download the clean data. Three tools (SOAP, Bowtie 2 and BWA) have been provided for reads mapping to the reference database; (ii) contig/scaffold/gene, sequences are compared with the selected database using blastn; and (iii) protein, sequences are compared with the selected database using tblastn.

The swine pathogen screening tool and workflows provide a user-friendly web-based interface, as shown in Figure 5. The users are required to submit their files into the system, select a specific tool or workflow and set the essential parameters. The submitted job gets incorporated into the tasks queue. If enough computing resources are not available, the job is placed on a waiting schedule. The task page is refreshed to update users on the status of the submitted jobs. When the job is complete, the results can be downloaded and visualized online. The user guide can be found at http://spdatabase.com:2080/faq.html.

### Validation of the swine pathogen screening tool

To evaluate the performance of the swine pathogen screening tool, we constructed 21 mock communities; the key results are listed in Table 1. We observed a strongly positive (r > 0.80) and highly significant (*P* < 0.01) Pearson correlation between the predicted and known proportions, indicating the accuracy of the tool. For read mapping to the reference database, SOAP, Bowtie 2 and BWA were used. The test results showed that SOAP runs faster than the other tools.

**Table 1:** Performance of the pathogen screening tool

Dataset number	Data type	Content	Data size	Analysis tool	Database	CPU	Running time	Correlation	*P*-value
1	Paired-end reads	Bacteria	5.43 × 10^9^	Bowtie 2	Bacteria	16	327	9.91 × 10^−1^	2.75 × 10^−8^
				BWA		8	435	9.91 × 10^−1^	2.56 × 10^−8^
				SOAP		8	225	9.94 × 10^−1^	6.29 × 10^−9^
2	Paired-end reads	Parasite	5.59 × 10^9^	Bowtie 2	Parasite	16	192	9.94 × 10^−1^	6.29 × 10^−9^
				BWA		8	205	9.64 × 10^−1^	7.26 × 10^−6^
				SOAP		8	233	9.82 × 10^−1^	4.53 × 10^−7^
3	Paired-end reads	Phage	5.67 × 10^9^	Bowtie 2	Phage	16	100	9.71 × 10^−1^	2.88 × 10^−6^
				BWA		8	89	9.71 × 10^−1^	2.93 × 10^−6^
				SOAP		8	57	9.75 × 10^−1^	1.77 × 10^−6^
4	Paired-end reads	Virus	4.26 × 10^9^	Bowtie 2	Virus	16	89	9.46 × 10^−1^	3.46 × 10^−5^
				BWA		8	119	9.41 × 10^−1^	4.89 × 10^−5^
				SOAP		8	59	9.60 × 10^−1^	1.07 × 10^−5^
5	Paired-end reads	Combine^*^	7.20 × 10^9^	Bowtie 2	Host and bacteria	26	1135	8.76 × 10^−1^	1.85 × 10^−4^
					Host and Parasite		1068		
					Host and phage		1077		
					Host and virus		1076		
				BWA	Host and bacteria	20	1485	8.76 × 10^−1^	1.86 × 10^−4^
					Host and parasite		1251		
					Host and phage		1269		
					Host and virus		1247		
				SOAP	Host and bacteria	20	631	8.13 × 10^−1^	1.31 × 10^−3^
					Host and parasite		528		
					Host and phage		563		
					Host and virus		510		
6	Contig	Bacteria	1.67 × 10^8^	blastn	Bacteria	20	49	1.00	6.65 × 10^−64^
7	16S tag	Bacteria	5.18 × 10^5^	blastn	16S	20	16	1.00	6.65 × 10^−64^
8	Gene	Bacteria	6.16 × 10^4^	blastn	Bacteria	20	14	9.82 × 10^−1^	4.19 × 10^−7^
9	Protein	Bacteria	2.03 × 10^4^	tblastn	Bacteria	20	21	9.08 × 10^−1^	2.85 × 10^−4^
10	Contig	Parasite	1.26 × 10^7^	blastn	Parasite	20	26	1.00	6.65 × 10^−64^
11	Gene	Parasite	6.00 × 10^4^	blastn	Parasite	20	14	9.95 × 10^−1^	3.50 × 10^−9^
12	Protein	Parasite	1.90 × 10^4^	tblastn	Parasite	20	16	9.87 × 10^−1^	1.16 × 10^−7^
13	Contig	Phage	2.62 × 10^6^	blastn	Phage	20	14	9.33 × 10^−1^	7.92 × 10^−5^
14	Gene	Phage	3.42 × 10^4^	blastn	Phage	20	16	9.31 × 10^−1^	9.05 × 10^−5^
15	Protein	Phage	1.14 × 10^4^	tblastn	Phage	20	17	8.58 × 10^−1^	1.50 × 10^−3^
16	Contig	Virus	2.12 × 10^6^	blastn	Virus	20	14	1.00	6.65 × 10^−64^
17	Gene	Virus	5.37 × 10^4^	blastn	Virus	20	13	9.33 × 10^−1^	7.92 × 10^−5^
18	Protein	Virus	1.79 × 10^4^	tblastn	Virus	20	14	9.57 × 10^−1^	1.36 × 10^−5^
19	Contig	Combine	1.02 × 10^8^	blastn	Bacteria	20	37	1.00	0.00
					Parasite		17		
					Phage		31		
					Virus		17		
20	Gene	Combine	9.50 × 10^4^	blastn	Bacteria	20	19	9.32 × 10^−1^	9.90 × 10^−6^
					Parasite		19		
					Phage		19		
					Virus		19		
21	Protein	Combine	3.04 × 10^4^	tblastn	Bacteria	20	27	8.16 × 10^−1^	1.22 × 10^−3^
					Parasite		19		
					Phage		21		
					Virus		17		

Ten columns are displayed. ‘Dataset number’ indicates the serial number of the resulting dataset. ‘Data type’ indicates the type of data displayed. ‘Content’ shows the species composition of the dataset, where ‘Combine’ indicates that the dataset contains sequences from bacteria, virus, parasite and phage, and asterisks (^*^) indicate that the dataset contains sequences from swine. ‘Data size’ indicates the number of bases or amino acids in the data. ‘Analysis tool’ lists the tool used for analysis. ‘Database’ lists the reference database used. ‘CPU’ lists the number of CPUs employed for the job. ‘Running time’ lists the total time taken for the job in minutes. ‘Correlation’ lists the average Pearson correlation coefficient between predicted and known relative proportions of species in the datasets. ‘P-value’ displays the observed P-values for analyzing differences between the predicted and known relative proportions of species. The data under ‘Data size’, ‘Correlation’ and ‘P-value’ are displayed in scientific notation.

### Case study: accurate detection of swine pathogens from clinical samples using the SPDB

To further validate the reliability of the application of the swine pathogen screening tool in detecting swine pathogens, two datasets were conducted to search for swine pathogens by using this online analysis platform (Supplementary Table S1). In the first dataset, composed of two NGS data from different piglets that died from CT, 13 viruses were successfully detected by mapping the raw reads to the swine virus references collected in this database (Supplementary Figure S1), including *Atypical porcine pestivirus* (APPV), known as the pathogen causing CT in piglets (35). The presence of APPV in the piglets was further confirmed by reverse transcription-PCR. Among the viruses identified in the NGS raw reads, PEDV, APPV, *Torque teno sus virus 1b* and *Sapelovirus A* were also found in the assembled scaffolds with lengths longer than 500 bp (Supplementary Table S2). The second dataset was used to search for potential pathogens that caused abortion in swine, including viral, bacterial and parasitic pathogens. Consistent with the previous PCR results, no viral pathogens associated with swine abortion were detected in the two stillborn fetuses. Similarly, though several bacterial pathogens were detected, none was considered as the pathogen that caused abortion in swine (Supplementary Figure S2A). Meanwhile, *Neospora caninum*, the newly recognized parasitic pathogen that can cause fetal mummification in swine (36), was detected with high relative abundance in both stillborn fetuses and further confirmed by real-time quantitative PCR (qPCR) (Supplementary Figure S2B). This suggested that the abortion in the selected clinical case may have been caused by this parasitic pathogen. The above results indicated the swine pathogen screening tool could provide a comprehensive analysis of swine pathogens in NGS data and thus was a powerful tool for swine disease diagnosis.

### Standard operating procedure

Since no generally accepted ‘analysis standard’ exists for NGS analysis workflow in swine disease diagnosis, the SPDB provides recommended parameters for every workflow of the pathogen screening tool. These recommended modes are regarded as SOPs related to the swine pathogen taxonomic classification. The modes are based on our laboratory experience and related studies (37–40). All parameters and steps included in these SOPs are subjected to strict checks and repeated validation.

### Resource integration

The SPDB was primarily constructed to support the application of NGS in swine disease diagnostics. In addition, it is important to combine NGS technology with clinical signs and symptoms and with confirmation by other diagnostic methods. Thus, we provided a quick link to access a remote diagnosis platform for major epidemics in pigs, which was created and is maintained by the Institute of Animal Health, Guangdong Academy of Agricultural Sciences. This platform was developed to provide a comprehensive remote diagnosis service for swine disease and realize online real-time visualization of swine disease data. By employing such huge data collection, we can efficiently apply big data and cloud computing technologies, analyze the occurrence rules of important swine diseases and predict the development trends and regional distributions of epidemics.

The SPDB also provides quick links to access other databases. Users can easily visit NCBI, DDBJ and European Molecular Biology Laboratory (EMBL). Databases of important pathogens can also be accessed, including the European Classical Swine Fever Virus Database (10), Influence Research Database (unpublished) and Foot and Mouth Disease Database (24). Pigs are important models for evolutionary research and are commonly used as biomedical models for studying reproduction, tissue degeneration/biological maintenance, stem cells and immune responses (41, 42). Thus, the links to addressable databases of swine have also been provided. The SUS-BAR is a database of pig proteins with statistically validated structural and functional annotation (43). PigVar is a database of pig variations and positive selection signatures (44). The porcine translational research database is a manually curated, genomics- and proteomics-based research resource (45). Lastly, LncRNAnet is a comprehensive *Sus scrofa* long non-coding RNA database (46).

## Discussion

Unbiased NGS approaches enable comprehensive pathogen detection in clinical microbiology laboratories and have numerous other applications in public health surveillance, outbreak investigation and infectious disease diagnosis. NGS technology is among the most promising strategy for the diagnosis of swine diseases; however, the lack of professional bioinformatics analysts in most laboratories limits its use for pathogen identification. Thus, we established the SPDB to implement more feasible NGS applications in the clinical diagnosis of swine diseases.

Before the SPDB was established, we collected and arranged swine pathogen information. The book *Diseases of Swine* has been thoroughly reviewed and published in 11 English editions and 10 Chinese editions (33), but updating a book is difficult and time-consuming. In 2015, a database of 137 pig infectious agents was constructed, which included their taxonomic divisions, zoonotic potential, status as emerging pathogens and information on OIE listings (47). The database, however, was not accessible. It is crucial to update pathogen information in real-time; thus, we established an up-to-date, comprehensive, convenient and freely available swine pathogen information database. We collected information on swine pathogens not only from the *Diseases of Swine* (33) but also from PubMed, Web of Science, the OIE official website and user submissions. In the first version of the SPDB, we collected information on 333 pathogens that were combined with pathogen genome data in a data search and download system. In the next version of the SPDB, we aim to provide more information on the pathogens, such as important studies, prevention and control measures, and create an independent swine pathogen information search system.

The SPDB is the first integrated database for swine pathogens and their related species. The SPDB has two main functions: a management system of sequences and a web-based analysis platform for swine pathogens. As a management system, 26 148 genome sequences of swine, bacterial pathogens, viral pathogens, parasitic pathogens and phage were collected, which are searchable in three modes using 17 fields. The SPDB genome search tool offers many advantages over the widely used sequence databases, such as NCBI (20), DDBJ (21), EMBL-ENA (22) and other pathogen-specific sequence databases (10, 23, 24). Firstly, compared with the other databases that only provide the English version, the SPDB is available in both English and Chinese, which is beneficial to the vast number of users whose native language is Chinese. Secondly, the SPDB is an easy-to-use specialized database that automates the core tasks in NGS data analysis of swine pathogens, while providing both the genomic and background information of pathogens. The SPDB also provides links to several addressable databases of swine to support academic exchange and improve database utilization. As a web-based analysis platform for swine pathogens, we designed a user-friendly system to achieve one-stop data management and data analysis services. In this version of SPDB, we developed a simple, standardized and rapid analysis pipeline from the receipt of clinical samples to the generation of a test report. This pipeline, named the swine pathogen screening tool, can identify 4403 swine pathogens and related species in clinical samples based on targeted 16S rRNA gene sequences and mNGS sequences. Our pipeline has several advantages: (i) it is a specific swine pathogen analysis tool; (ii) a range of data types can be accepted, including reads, contig, scaffold, gene, protein and 16S rRNA; (iii) the customized reference databases can save computing resources as well as reduce computing time; and (iv) thorough visual results are provided. The analysis results of 21 different mock communities and 2 datasets of clinical samples indicated that the swine pathogen screening tool was a powerful tool for swine disease diagnosis. However, this tool provides only a prediction of the suspected pathogens and hence orthogonal traditional methods such as culturing, qPCR and serologic assays used for routine clinical diagnosis of swine disease are still required to confirm the diagnosis.

The database and workflows are frequently updated to reflect the availability of new datasets and studies. The aim is to establish a ‘one-click-analysis’ pipeline for pathogen identification in complex samples using NGS to provide a rapid and accurate tool for veterinarians and researchers. The future version of the SPDB will provide predefined workflows for targeted 16S rRNA gene sequencing, mNGS and WGS. These workflows will contain guidelines for sampling and sequencing, raw reads preprocessing, sequence assembly, genome structural analysis, database annotation, community profiling and sequence alignment. Additionally, we aim to set up SOPs and standards for NGS workflows for the rapid diagnosis of swine pathogens. The standardization will enable efficient data sharing, improve the transparency of data collection and analysis and facilitate meta-analyses. Such predefined workflows, however, are not ideal for all analyses. Therefore, the future version of the SPDB will provide customized workflows for the professional bioinformaticians interested in the method or workflow development, encouraging them to share their developments on our platform. The SPDB is willing to provide an open-source for swine disease research.

Current metagenomics data and associated analysis results are widely dispersed around different types of resources from public data archives to specialized databases. Integration of the varied types of metagenomics data is essential to overcome major obstacles in data reusability, accessibility and transparency. Prior to our work, no specialized metagenomics database had been developed for swine pathogens. This study will pave the way for the development of a curated database for swine metagenomics data (including both 16S and metagenomics sequencing data) providing a clear, concise and up-to-date overview of the differences in microbial composition across the different swine diseases, together with relevant information on the published studies. The effective management of swine metagenomics data and associated analysis results will contribute to the application of NGS in swine health and disease research.

## Conclusion

The SPDB is the first integrated swine pathogen database and first web-based NGS data analysis platform for swine pathogens. The primary mission of the SPDB is to facilitate the analysis and management of datasets for researchers with a user-friendly web interface. Currently, the SPDB provides two powerful tools: an online genome search tool covering 26 148 genomes and a swine pathogen screening tool covering 4403 swine pathogens and related species. With the generation and integration of more advanced data, the SPDB could be potentially developed into a global swine pathogen database and data analysis platform for swine diseases.

## Supplementary data

Supplementary data are available at *Database* online.

## Funding

National Key Basic Research Program (2016YFD0500606); Natural Science Foundation of China (31800105); Natural Science Foundation of Guangdong Province (2018A030310486); ‘Climbing plan’ supported by Guangdong University students’ Special Fund for Scientific and Technological Innovation and Cultivation (pdjh2020b0043); Guangdong Provincial Key Research and Development Plan Project (2019B020212002).

## Conflict of interest

Authors have no conflict of interest to declare.

## Supplementary Material

suppl_data_baaa063

## References

[1] Lu G., Cai S. and Zhang G. (2019) African swine fever in China one year on. Vet. Rec., 185, 542. 10.1136/vr.l6238 31676618

[2] Vergne T., Chen-Fu C., Li S. et al. (2017) Pig empire under infectious threat: risk of African swine fever introduction into the People’s Republic of China. Vet. Rec., 181, 117. 28754737 10.1136/vr.103950

[3] Wang T., Sun Y. and Qiu H. (2018) African swine fever: an unprecedented disaster and challenge to China. Infect. Dis. Poverty, 7, 111. 30367672 10.1186/s40249-018-0495-3 PMC6203974

[4] Lee C. (2015) *Porcine epidemic diarrhea virus*: an emerging and re-emerging epizootic swine virus. Virol. J., 12, 193. 26689811 10.1186/s12985-015-0421-2 PMC4687282

[5] Guo Z., Chen X., Li R. et al. (2018) The prevalent status and genetic diversity of *Porcine reproductive and respiratory syndrome virus* in China: a molecular epidemiological perspective. Virol. J., 15, 2. 29301547 10.1186/s12985-017-0910-6 PMC5753475

[6] Williamson S. (2018) *Streptococcus suis* disease in pigs. Vet. Rec., 183, 408–410. 30287563 10.1136/vr.k4181

[7] Maes D., Sibila M., Kuhnert P. et al. (2018) Update on *Mycoplasma hyopneumoniae* infections in pigs: knowledge gaps for improved disease control. Transbound. Emerg. Dis., 65, 110–124. 28834294 10.1111/tbed.12677

[8] Stelzer S., Basso W., Benavides S.J. et al. (2019) *Toxoplasma gondii* infection and toxoplasmosis in farm animals: risk factors and economic impact. Food Waterborne Parasitol., 15, e37. 10.1016/j.fawpar.2019.e00037 PMC7033994 32095611

[9] Bhattarai R., Carabin H., Proaño J.V. et al. (2019) The monetary burden of cysticercosis in Mexico. PLoS Negl. Trop. Dis., 13, e7501. 10.1371/journal.pntd.0007501 PMC6645581 31291239

[10] Postel A., Schmeiser S., Zimmermann B. et al. (2016) The European Classical Swine Fever Virus Database: blueprint for a pathogen-specific sequence database with integrated sequence analysis tools. Viruses, 8, 302. 10.3390/v8110302 PMC5127016 27827988

[11] Simner P.J., Miller S. and Carroll K.C. (2018) Understanding the promises and hurdles of metagenomic next-generation sequencing as a diagnostic tool for infectious diseases. Clin. Infect. Dis., 66, 778–788. 29040428 10.1093/cid/cix881 PMC7108102

[12] Balloux F., Brønstad B.O., van Dorp L. et al. (2018) From theory to practice: translating whole-genome sequencing (WGS) into the clinic. Trends Microbiol., 26, 1035–1048. 30193960 10.1016/j.tim.2018.08.004 PMC6249990

[13] Bender J.M., Li F., Adisetiyo H. et al. (2018) Quantification of variation and the impact of biomass in targeted 16S rRNA gene sequencing studies. Microbiome, 6, 155. 30201048 10.1186/s40168-018-0543-z PMC6131952

[14] Nilsson R.H., Anslan S., Bahram M. et al. (2019) Mycobiome diversity: high-throughput sequencing and identification of fungi. Nat. Rev. Microbiol., 17, 95–109. 30442909 10.1038/s41579-018-0116-y

[15] Cannon M.V., Bogale H., Rutt L. et al. (2018) A high-throughput sequencing assay to comprehensively detect and characterize unicellular eukaryotes and helminths from biological and environmental samples. Microbiome, 6, 195. 30373673 10.1186/s40168-018-0581-6 PMC6206884

[16] Chen Q., Wang L., Zheng Y. et al. (2018) Metagenomic analysis of the RNA fraction of the fecal virome indicates high diversity in pigs infected by porcine endemic diarrhea virus in the United States. Virol. J., 15, 95. 29801460 10.1186/s12985-018-1001-z PMC5970503

[17] Zhou P., Fan H., Lan T. et al. (2018) Fatal swine acute diarrhoea syndrome caused by an HKU2-related coronavirus of bat origin. Nature, 556, 255–258. 29618817 10.1038/s41586-018-0010-9 PMC7094983

[18] Wang Q., Cai R., Huang A. et al. (2018) Comparison of oropharyngeal microbiota in healthy piglets and piglets with respiratory disease. Front. Microbiol., 9, 3218. 30627125 10.3389/fmicb.2018.03218 PMC6309737

[19] Huang A., Cai R., Wang Q. et al. (2019) Dynamic change of gut microbiota during *Porcine epidemic diarrhea virus* infection in suckling piglets. Front. Microbiol., 10, 322. 30858839 10.3389/fmicb.2019.00322 PMC6397872

[20] Sayers E.W., Beck J., Brister J.R. et al. (2020) Database resources of the National Center for Biotechnology Information. Nucleic Acids Res., 48, D9–D16. 31602479 10.1093/nar/gkz899 PMC6943063

[21] Ogasawara O., Kodama Y., Mashima J. et al. (2019) DDBJ database updates and computational infrastructure enhancement. Nucleic Acids Res., 48, D45–D50. 10.1093/nar/gkz982 PMC7145692 31724722

[22] Amid C., Alako B.T.F., Balavenkataraman K.V. et al. (2019) The European Nucleotide Archive in 2019. Nucleic Acids Res., 48, D70–D76. 10.1093/nar/gkz1063 PMC7145635 31722421

[23] Stalder H., Hug C., Zanoni R. et al. (2016) A nationwide database linking information on the hosts with sequence data of their virus strains: a useful tool for the eradication of bovine viral diarrhea (BVD) in Switzerland. Virus Res., 218, 49–56. 26403669 10.1016/j.virusres.2015.09.012

[24] Knowles N.J. and Samuel A.R. (2003) Molecular epidemiology of *Foot-and-mouth disease virus*. Virus Res., 91, 65–80. 12527438 10.1016/s0168-1702(02)00260-5

[25] Meyer F., Paarmann D., D'Souza M. et al. (2008) The metagenomics RAST server—a public resource for the automatic phylogenetic and functional analysis of metagenomes. BMC Bioinformatics, 9, 386. 18803844 10.1186/1471-2105-9-386 PMC2563014

[26] Roux S., Tournayre J., Mahul A. et al. (2014) Metavir 2: new tools for viral metagenome comparison and assembled virome analysis. BMC Bioinformatics, 15, 76. 24646187 10.1186/1471-2105-15-76 PMC4002922

[27] Dhariwal A., Chong J., Habib S. et al. (2017) MicrobiomeAnalyst: a web-based tool for comprehensive statistical, visual and meta-analysis of microbiome data. Nucleic Acids Res., 45, W180–W188. 28449106 10.1093/nar/gkx295 PMC5570177

[28] Glöckner F.O., Yilmaz P., Quast C. et al. (2017) 25 years of serving the community with ribosomal RNA gene reference databases and tools. J. Biotechnol., 261, 169–176. 28648396 10.1016/j.jbiotec.2017.06.1198

[29] Zhang T., Miao J., Han N. et al. (2018) MPD: a pathogen genome and metagenome database. Database, 2018, 1–6. 10.1093/database/bay055 PMC6007212 29917040

[30] Sinha R., Abu-Ali G., Vogtmann E. et al. (2017) Assessment of variation in microbial community amplicon sequencing by the Microbiome Quality Control (MBQC) project consortium. Nat. Biotechnol., 35, 1077–1086. 28967885 10.1038/nbt.3981 PMC5839636

[31] Anonymous (2017) Overcoming hurdles in sharing microbiome data. Nat. Microbiol., 2, 1573. 29176696 10.1038/s41564-017-0077-3

[32] Costea P.I., Zeller G., Sunagawa S. et al. (2017) Towards standards for human fecal sample processing in metagenomic studies. Nat. Biotechnol., 35, 1069–1076. 28967887 10.1038/nbt.3960

[33] Zimmerman J., Karriker L., Ramirez A. et al. (2019) Diseases of Swine, 11th edn. Wiley-Blackwell, Hoboken, pp. 425–1040.

[34] Bankevich A., Nurk S., Antipov D. et al. (2012) SPAdes: a new genome assembly algorithm and its applications to single-cell sequencing. J. Comput. Biol., 19, 455–477. 22506599 10.1089/cmb.2012.0021 PMC3342519

[35] Gatto I., Sonalio K. and de Oliveira L.G. (2019) *Atypical Porcine pestivirus* (APPV) as a new species of *Pestivirus* in pig production. Front. Vet. Sci., 6, 35. 30847345 10.3389/fvets.2019.00035 PMC6393339

[36] Snak A., Serighelli G. Jr., Pilati G. et al. (2019) Does *Neospora caninum* cause reproductive problems in pigs? Vet. Parasitol., 275, 108934. 31600613 10.1016/j.vetpar.2019.108934

[37] Li R., Li Y., Kristiansen K. et al. (2008) SOAP: short oligonucleotide alignment program. Bioinformatics, 24, 713–714. 18227114 10.1093/bioinformatics/btn025

[38] Li H. and Durbin R. (2009) Fast and accurate short read alignment with Burrows–Wheeler transform. Bioinformatics, 25, 1754–1760. 19451168 10.1093/bioinformatics/btp324 PMC2705234

[39] Langmead B. and Salzberg S.L. (2012) Fast gapped-read alignment with Bowtie 2. Nat. Methods, 9, 357–359. 22388286 10.1038/nmeth.1923 PMC3322381

[40] Mount D.W. (2007) Using the Basic Local Alignment Search Tool (BLAST). CSH Protoc., 2007, p17. 10.1101/pdb.top17 21357135

[41] Fang M., Hu X., Jiang T. et al. (2005) The phylogeny of Chinese indigenous pig breeds inferred from microsatellite markers. Anim. Genet., 36, 7–13. 15670125 10.1111/j.1365-2052.2004.01234.x

[42] Vodicka P., Smetana K.J., Dvorankova B. et al. (2005) The miniature pig as an animal model in biomedical research. Ann. N. Y. Acad. Sci., 1049, 161–171. 15965115 10.1196/annals.1334.015

[43] Piovesan D., Profiti G., Martelli P.L. et al. (2013) SUS-BAR: a database of pig proteins with statistically validated structural and functional annotation. Database, 2013, t65. 10.1093/database/bat065 PMC3781388 24065691

[44] Zhou Z.Y., Li A., Otecko N.O. et al. (2017) PigVar: a database of pig variations and positive selection signatures. Database, 2017, 1–10. 10.1093/database/bax048 PMC5502369 29220438

[45] Dawson H.D., Chen C., Gaynor B. et al. (2017) The porcine translational research database: a manually curated, genomics and proteomics-based research resource. BMC Genomics, 18, 643. 28830355 10.1186/s12864-017-4009-7 PMC5568366

[46] Liang G., Yang Y., Li H. et al. (2018) LncRNAnet: a comprehensive *Sus scrofa* lncRNA database. Anim. Genet., 49, 632–635. 30276831 10.1111/age.12720

[47] Díaz I., Cortey M., Olvera À. et al. (2016) Use of H-index and other Bibliometric indicators to evaluate research productivity outcome on swine diseases. PLoS One, 11, e149690. 10.1371/journal.pone.0149690 PMC4773010 26930283

