# Supplementary material for: SPDB: a specialized database and web-based analysis platform for swine pathogens
Source: Database (Oxford). 2020 Aug 6;2020:baaa063. doi: 10.1093/database/baaa063 (PMC7409514; doi:10.1093/database/baaa063)
Supplement: suppl_data_baaa063 [file suppl_data_baaa063.zip › Supplementary Table S1.docx]

Supplementary Table S1. Statistical table of datasets.

| Dataset ID | Sample ID | Clean reads | Clean reads (remove host) | Scaffolds |
| --- | --- | --- | --- | --- |
| 1 | H2F | 80,195,598 | 4,333,380 | 30,827 |
|  | YJ2 | 47,231,468 | 2,574,792 | 3,767 |
| 2 | JX4 | 49,968,206 | 158,102 | 3,293 |
|  | JX5 | 38,557,008 | 91,618 | 2,575 |
