# Supplementary material for: SPDB: a specialized database and web-based analysis platform for swine pathogens
Source: Database (Oxford). 2020 Aug 6;2020:baaa063. doi: 10.1093/database/baaa063 (PMC7409514; doi:10.1093/database/baaa063)
Supplement: suppl_data_baaa063 [file suppl_data_baaa063.zip › Supplementary Table S2.docx]

Supplementary Table S2. The analysis result of the scaffolds of the first dataset.

| Sample ID | Query id | Subject id | Identity | Align length | Mismatch | Gap | Query start | Query end | Subject start | Subject end | E value | Score | Subject length | Species |
| --- | --- | --- | --- | --- | --- | --- | --- | --- | --- | --- | --- | --- | --- | --- |
| H2F | NODE_10_length_2390_cov_6.469379 | KC210147.1 | 99.916 | 2390 | 2 | 0 | 1 | 2390 | 9992 | 12381 | 0 | 4403 | 28037 | *Porcine epidemic diarrhea virus* |
|  | NODE_110_length_827_cov_7.367876 | MF462814.1 | 100 | 827 | 0 | 0 | 1 | 827 | 13921 | 14747 | 0 | 1528 | 28063 | *Porcine epidemic diarrhea virus* |
|  | NODE_112_length_822_cov_5.166884 | MG837011.1 | 100 | 820 | 0 | 0 | 3 | 822 | 15384 | 16203 | 0 | 1515 | 28038 | *Porcine epidemic diarrhea virus* |
|  | NODE_114_length_812_cov_6.483487 | KM225244.1 | 99.754 | 812 | 2 | 0 | 1 | 812 | 1378 | 2189 | 0 | 1489 | 4155 | *Porcine epidemic diarrhea virus* |
|  | NODE_121_length_773_cov_8.537604 | MF782686.1 | 99.855 | 689 | 1 | 0 | 3 | 691 | 5091 | 5779 | 0 | 1267 | 28010 | *Porcine epidemic diarrhea virus* |
|  | NODE_123_length_771_cov_6.543296 | KF840551.1 | 99.87 | 771 | 1 | 0 | 1 | 771 | 5233 | 6003 | 0 | 1419 | 7614 | *Porcine epidemic diarrhea virus* |
|  | NODE_137_length_737_cov_5.448680 | KX354742.1 | 93.08 | 737 | 51 | 0 | 1 | 737 | 4526 | 5262 | 0 | 1079 | 6996 | *Sapelovirus A* |
|  | NODE_15_length_1868_cov_7.215113 | KM609207.1 | 99.893 | 1868 | 2 | 0 | 1 | 1868 | 3590 | 1723 | 0 | 3439 | 28050 | *Porcine epidemic diarrhea virus* |
|  | NODE_150_length_721_cov_5.609610 | MH013466.1 | 100 | 721 | 0 | 0 | 1 | 721 | 3819 | 4539 | 0 | 1332 | 27667 | *Porcine epidemic diarrhea virus* |
|  | NODE_19_length_1787_cov_5.714781 | MF462814.1 | 99.832 | 1787 | 3 | 0 | 1 | 1787 | 20731 | 18945 | 0 | 3284 | 28063 | *Porcine epidemic diarrhea virus* |
|  | NODE_200_length_655_cov_14.966667 | MG799362.1 | 98.931 | 655 | 7 | 0 | 1 | 655 | 1523 | 869 | 0 | 1171 | 2064 | *Torque teno sus virus 1b* |
|  | NODE_276_length_592_cov_5.698324 | KU975389.1 | 100 | 592 | 0 | 0 | 1 | 592 | 13532 | 12941 | 0 | 1094 | 28044 | *Porcine epidemic diarrhea virus* |
|  | NODE_289_length_584_cov_21.330813 | JX088695.1 | 99.658 | 584 | 1 | 1 | 2 | 584 | 28038 | 27455 | 0 | 1066 | 28038 | *Porcine epidemic diarrhea virus* |
|  | NODE_34_length_1418_cov_7.134263 | KM609207.1 | 99.859 | 1418 | 2 | 0 | 1 | 1418 | 1714 | 297 | 0 | 2608 | 28050 | *Porcine epidemic diarrhea virus* |
|  | NODE_355_length_541_cov_6.427984 | MF440660.1 | 94.834 | 542 | 27 | 1 | 1 | 541 | 17 | 558 | 0 | 845 | 7227 | *Sapelovirus A* |
|  | NODE_388_length_524_cov_6.552239 | MH107322.1 | 99.809 | 524 | 1 | 0 | 1 | 524 | 6097 | 6620 | 0 | 963 | 28038 | *Porcine epidemic diarrhea virus* |
|  | NODE_435_length_507_cov_7.933628 | JX286666.1 | 98.419 | 506 | 8 | 0 | 1 | 506 | 7517 | 7012 | 0 | 891 | 7572 | *Sapelovirus A* |
|  | NODE_451_length_502_cov_3.487696 | MK347476.1 | 100 | 502 | 0 | 0 | 1 | 502 | 1363 | 862 | 0 | 928 | 11043 | *Atypical porcine pestivirus* |
|  | NODE_456_length_500_cov_5.826966 | MF782687.1 | 99.6 | 500 | 2 | 0 | 1 | 500 | 22861 | 23360 | 0 | 913 | 28038 | *Porcine epidemic diarrhea virus* |
|  | NODE_62_length_1122_cov_6.248360 | MG837011.1 | 99.911 | 1122 | 1 | 0 | 1 | 1122 | 7023 | 8144 | 0 | 2067 | 28038 | *Porcine epidemic diarrhea virus* |
|  | NODE_69_length_1077_cov_6.849315 | KX354743.1 | 94.429 | 1077 | 60 | 0 | 1 | 1077 | 6445 | 5369 | 0 | 1657 | 6999 | *Sapelovirus A* |

Supplementary Table S2 Continued

| Sample ID | Query id | Subject id | Identity | Align length | Mismatch | Gap | Query start | Query end | Subject start | Subject end | E value | Score | Subject length | Species |
| --- | --- | --- | --- | --- | --- | --- | --- | --- | --- | --- | --- | --- | --- | --- |
| H2F | NODE_75_length_1045_cov_9.392929 | MF440645.1 | 91.587 | 1046 | 86 | 2 | 1 | 1045 | 2867 | 3911 | 0 | 1443 | 7258 | *Sapelovirus A* |
|  | NODE_84_length_958_cov_6.477298 | MH107322.1 | 99.896 | 958 | 1 | 0 | 1 | 958 | 9967 | 9010 | 0 | 1764 | 28038 | *Porcine epidemic diarrhea virus* |
|  | NODE_9_length_2418_cov_5.654253 | MF462814.1 | 99.917 | 2418 | 2 | 0 | 1 | 2418 | 16402 | 18819 | 0 | 4455 | 28063 | *Porcine epidemic diarrhea virus* |
|  | NODE_91_length_906_cov_9.708578 | MF440651.1 | 90.066 | 906 | 89 | 1 | 2 | 906 | 1418 | 2323 | 0 | 1173 | 7221 | *Sapelovirus A* |
|  | NODE_96_length_879_cov_11.123786 | KM609207.1 | 99.544 | 878 | 4 | 0 | 1 | 878 | 26364 | 27241 | 0 | 1600 | 28050 | *Porcine epidemic diarrhea virus* |
